# Peptidome profiling dataset of ovarian cancer and non-cancer proximal fluids: Ascites and blood sera

**DOI:** 10.1016/j.dib.2018.12.056

**Published:** 2018-12-19

**Authors:** Victoria Shender, Georgij Arapidi, Ivan Butenko, Nikolay Anikanov, Olga Ivanova, Vadim Govorun

**Affiliations:** a Federal Research and Clinical Center of Physical-Chemical Medicine of the Federal Medical and Biological Agency of the Russian Federation, Malaya Pirogovskaya 1a, Moscow 119435, Russian Federation; b Shemyakin-Ovchinnikov Institute of Bioorganic Chemistry of the Russian Academy of Sciences, Miklukho-Maklaya str. 16/10, Moscow 117997, Russian Federation; c Moscow Institute of Physics and Technology (State University), Institutskii Per. 9, Moscow Region, Dolgoprudny 141700, Russian Federation

## Abstract

Despite a large number of proteomic studies of biological fluids from ovarian cancer patients, there is a lack of sensitive screening methods in clinical practice (Kim et al., 2016) (DOI:https://doi.org/10.1111/cas.12987[1]). Low molecular weight endogenous peptides more easily diffuse across endothelial barriers than proteins and can be more relevant biomarker candidates (Meo et al., 2016) (DOI:https://doi.org/10.18632/oncotarget.8931[2], (Bery et al., 2014) DOI:https://doi.org/10.1186/1559-0275-11-13[3], (Huang et al., 2018) DOI:https://doi.org/10.1097/IGC.0000000000001166[4]). Detailed peptidomic analysis of 26 ovarian cancer and 15 non-cancer samples of biological fluids (ascites and sera) were performed using TripleTOF 5600+ mass-spectrometer. Prior to LC-MS/MS analysis, peptides were extracted from biological fluids using anion exchange sorbent with subsequent peptide desorption from the surface of highly abundant proteins. In total, we identified 4874 peptides; 3123 peptides were specific for the ovarian cancer samples. The mass-spectrometry peptidomics data presented in this data article have been deposited to the ProteomeXchange Consortium (Deutsch et al., 2017) (DOI:https://doi.org/10.1093/nar/gkw936[5]) via the PRIDE partner repository with the dataset identifier PXD009382 and https://doi.org/10.6019/PXD009382, http://www.ebi.ac.uk/pride/archive/projects/PXD009382.

**Specifications table**

**Table:** 

Subject area	Biochemistry
More specific subject area	Peptidomics, Ovarian Cancer Biomarkers
Type of data	Table, LC-MS/MS data and identification data
How data were acquired	Peptides were analyzed on a TripleTOF 5600+ mass spectrometer with a NanoSpray III ion source (Sciex, Canada) coupled with a NanoLC Ultra 2D+ nano-HPLC system (Eksigent, USA)
Data format	Raw and analyzed data
Experimental factors	6 ovarian cancer and 5 cirrhosis ascites samples;
	Serum samples from 20 ovarian cancer patients;
	Serum samples from 10 healthy donors.
Experimental features	Pools of 6 ovarian cancer ascites, 5 cirrhosis ascites, 10 serum samples from healthy donors and two pools of 10 ovarian cancer sera were fractionated using anion exchange QAE Sephadex A-25 sorbent and analyzed by LC-MS/MS.
Data source location	Federal Research and Clinical Center of Physical Chemical Medicine of the Federal Medical and Biological Agency of the Russian Federation, Malaya Pirogovskaya 1a, Moscow 119435, Russian Federation.
Data accessibility	Data are with this article. The LC-MS/MS data have been deposited to the ProteomeXchange Consortium via the PRIDE [6] partner repository with the dataset identifier PXD009382 and 10.6019/PXD009382.
	Direct download link: http://www.ebi.ac.uk/pride/archive/projects/PXD009382

**Value of the data**

• The data describe the most detailed peptidome study of ascites from ovarian cancer patients after chemotherapy and from cirrhosis patients as well as peptides of sera from ovarian cancer patients and healthy donors. These data can be used for analysis of the action of cancer specific proteases.
• Specific ovarian cancer peptides from this dataset can be used in further studies to identify potential biomarkers.
• The data might be useful for detection of somatic mutations implicated in ovarian cancer after chemotherapy.
• The dataset allows for extended statistical analysis, and we encourage such collaborations.

## 1 Data

An endogenous peptide profile is presented from LC-MS/MS analysis of 11 ascites samples, 6 from ovarian cancer patients after chemotherapy and 5 from non-cancer patients with cirrhosis; and 30 serum samples, 20 from ovarian cancer patients and 10 from healthy donors.

Pools of ascites and serum samples were fractionated using anion exchange QAE Sephadex A-25 sorbent followed by peptide desorption from the surface of abundant blood plasma proteins [7] and analyzed by LC-MS/MS on Sciex TripleTOF 5600+ Q-TOF mass-spectrometer (Table 1, Supplementary Table S1).

**Table 1:** Characteristics of analyzed samples in each group.

**Biomaterial type**	**Pools**	**Number of samples**	**Number of replicates**
Ovarian Cancer Ascites	Pool 1	3 Ovarian Cancer patients after chemotherapy	2 replicates
	Pool 2	3 Ovarian Cancer patients after chemotherapy	2 replicates
Cirrhosis Ascites	Pool 1	5 female patients with Portal Alcoholic Cirrhosis	2 replicates
Ovarian Cancer Sera	Pool 1	10 Ovarian Cancer patients after chemotherapy	1 replicate
	Pool 2	10 Ovarian Cancer patients after chemotherapy	2 replicates
Healthy Donors’ Sera	Pool 1	10 female healthy donors	2 replicates

The LC-MS/MS raw data files are deposited to the ProteomeXchange Consortium under the dataset identifier PXD009382 (http://www.ebi.ac.uk/pride/archive/projects/PXD009382).

**Fig. 1:**
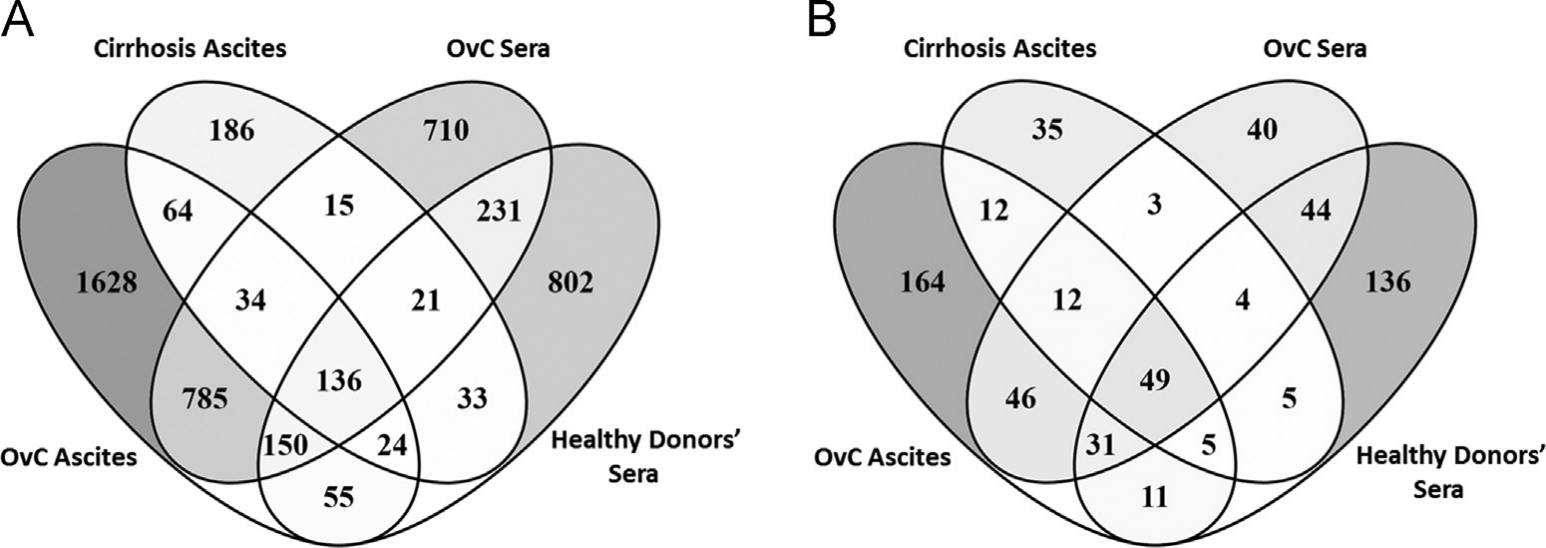
Distribution of (A) peptides and (B) precursor proteins identified in malignant and cirrhosis ascites and in sera from ovarian cancer patients and healthy donors. *OvC* – ovarian cancer.

Initial analysis allowed to identify 4874 unique endogenous peptides (Fig. 1A) derived from 597 proteins, 250 of which were found only in ovarian cancer samples (Fig. 1B). The distribution of the identified peptides by groups is shown in Table 2 and Supplementary Table S2.

**Table 2:** Number of identified peptides and precursor proteins.

**Sample group**	**Number of identified peptides**	**Number of precursor proteins**
Ovarian Cancer Ascites	2876	330
Cirrhosis Ascites	513	125
Ovarian Cancer Sera	2082	229
Healthy Donors׳ Sera	1452	285
Total Number	4874	597

Functional enrichment analysis of precursor proteins specific for ovarian cancer proximal fluids (ascites and sera) demonstrated that the most pronounced cluster was Ribosome (Supplementary Table S3).

## 2 Experimental design, materials and methods

### 2.1 Materials

QAE Sephadex A-25 (GE Healthcare Bio-Sciences AB, Sweden), C18 Discovery Supelco RP-SPE cartridge (50 mg; Sigma-Aldrich St. Louis, MO, USA). Formic acid (FA), Trifluoroacetic acid (TFA), and Sodium hydroxide were acquired from Sigma-Aldrich (St. Louis, MO, USA). LiChrosolv acetonitrile for LC-MS (AсN), LiChrosolv acetone for liquid chromatography, LiChrosolv methanol gradient grade for liquid chromatography (MeOH), LiChrosolv ethanol gradient grade for liquid chromatography and HPLC grade water were acquired from Merck (Darmstadt, Germany).

### 2.2 Patients and specimens

Ascitic samples from 6 ovarian cancer patients (“Malignant ascites”; average age 57 years) and serum samples from 20 ovarian cancer patients (average age 57 years) were obtained from the Russian Scientific Center of Roentgenoradiology (Moscow, Russia). Ascitic samples from 5 patients with portal alcoholic cirrhosis (hereinafter, “Cirrhosis ascites”; average age 57 years) were obtained from the Central Research Institute of Gastroenterology (Moscow, Russia).

Serum samples from 10 healthy female (average age 26 years) donors were collected from the Federal Research and Clinical Center of Physical Chemical Medicine of the Federal Medical and Biological Agency of the Russian Federation.

Characteristics of the biological material are given in Supplementary Table S1. All diagnoses were confirmed by morphological studies. The study was approved by the Ethics Committees of corresponding hospitals, and all the patients and volunteers gave written informed consent for their participation.

### 2.3 Sample collection

The ascitic fluids from both groups were taken in sterile standard tubes of 50-mL volume (Eppendorf). The ascitic fluids were centrifuged at 200 g for 15 min at room temperature in order to remove the cells. The samples were stored at −80 °C and transported in liquid nitrogen. To obtain serum, samples were collected from cubital vein into blood collection tubes (REF 6,456092 mL, Vacuette tube, Austria). Serum was obtained after coagulation of blood for 1 h at room temperature. The collection tubes were centrifuged for 15 min at 700 g at room temperature. The serum was separated from the clot, aliquoted and stored at −80 °C until analysis.

Prior to peptidomic analysis, the samples were centrifuged at 16,000 g for 30 min to remove the cellular debris.

### 2.4 Plasma and serum fractionation

Ascites and serum samples were fractionated on QAE Sephadex A-25 (GE Healthcare Bio-Sciences AB, Sweden) strong anion exchange particles as described previously [8]. Briefly, 80 µL of sorbent was washed 2 times with 400 µL Wash buffer 1 (20 mM Tris (pH = 8.26)). To precipitate the sorbent, the samples were centrifuged at 500 g for 10 s. Then the supernatant was accurately removed; 200 µL of ascites/serum was diluted with 400 µL Wash buffer 1 and added to the sorbent. After 30 min of incubation with vortexing, the sorbent was washed 3 times with 700 µL Wash buffer 1 and incubated with 800 µL of Elution buffer (0.5% TFA) for 15 min.

### 2.5 Peptide desorption from abundant proteins

To desorb peptides from the surface of highly abundant proteins, we used the technique described earlier [7], [8]. The eluates after anion exchange chromatography were incubated at 98 °C for 15 min. After heating, the samples were desalted using C18 Discovery Supelco RP-SPE cartridge (50 mg; Sigma-Aldrich St. Louis, MO, USA). Eluates were vacuum-dried to 5 µL, diluted with 10 µL 3% AcN, 0.1% TFA, and stored at −80 °C before LC–MS/MS analysis.

### 2.6 LC-MS/MS analysis

Analysis was performed on a TripleTOF 5600+ mass spectrometer with a NanoSpray III ion source (Sciex, Canada) coupled with a NanoLC Ultra 2D+ nano-HPLC system (Eksigent, USA) as described previously [8]. The HPLC system was configured in the trap-elute mode. For sample loading buffer and buffer A, a mixture of 98.9% water, 1% MeOH, 0.1% FA (v/v) was used. Buffer B was 99.9% AcN and 0.1% FA (v/v). Samples were loaded on a Chrom XP C18 trap column (3 µm, 120 Å, 350 µm 0.5 mm; Eksigent) at a flow rate of 3 µL/min for 10 min and eluted through a 3C18-CL-120 separation column (3 µm, 120 Å, 75 µm 150 mm; Eksigent) at a flow rate of 300 nL/min. The gradient was from 5% to 40% buffer B in 90 min followed by 10 min at 95% buffer B and 20 min of re-equilibration with 5% buffer B. Between different samples, two blank 45 min runs consisting of 5–8 min waves (5% B, 95%, 95%, 5%) were required to wash the system and to prevent carryover.

The information-dependent mass-spectrometer experiment included one survey MS1 scan followed by 50 dependent MS2 scans. MS1 acquisition parameters were as follows: the mass range for MS2 analysis was 300–1250 m/z, and the signal accumulation time was 250 ms. Ions for MS2 analysis were selected on the basis of intensity with a threshold of 200 counts per second and a charge state from 2 to 5. MS2 acquisition parameters were as follows: the resolution of the quadrupole was set to UNIT (0.7 Da), the measurement mass range was 200–1800 *m*/*z*, and the signal accumulation time was 50 ms for each parent ion. Collision-activated dissociation was performed with nitrogen gas with the collision energy ramped from 25 to 55 V within the signal accumulation time of 50 ms. Analyzed parent ions were sent to the dynamic exclusion list for 15 s in order to get an MS2 spectra at the chromatographic peak apex.

β-Galactosidase tryptic solution (20 fmol) was run with a 15-min gradient (5–25% buffer B) every two samples and between sample sets to calibrate the mass spectrometer and to control the overall system performance, stability, and reproducibility.

### 2.7 Peptide identification

Raw LC-MS/MS data were converted to.mgf peaklists with ProteinPilot (version 4.5, Sciex, Canada). For this procedure, we ran ProteinPilot in identification mode with the following parameters: no specific digestion, TripleTOF 5600 instrument, thorough ID search with detected protein threshold 95.0% against the UniProt human protein knowledgebase. For thorough protein identification, the generated peak lists were searched with the MASCOT (version 2.5.1, Matrix Science Ltd., UK) and X! Tandem (VENGEANCE, 2015.12.15, The Global Proteome Machine Organization) search engines against the UniProt human protein knowledgebase. The precursor and fragment mass tolerance were set at 20 ppm and 50 ppm, respectively. Database-searching parameters included the following: no specific digestion. For X! Tandem we also selected parameters that allowed quick check for protein N-terminal residue acetylation, peptide N-terminal glutamine ammonia loss or peptide N-terminal glutamic acid water loss. Resulting files were submitted to the Scaffold 4 software (version 4.2.1, Proteome Software, Inc., USA) for validation and further analysis. We used the local false discovery rate scoring algorithm with standard experiment-wide protein grouping. For the evaluation of peptide hits, a false discovery rate less than 1% was selected for peptides only. False positive identifications were based on reverse database analysis.

### 2.8 Functional enrichment analysis

Precursor proteins unique for ovarian cancer samples were analysed via STRING (Search Tool for the Retrieval of Interacting Genes/Proteins) website [9]. The list of the precursor protein identifies were uploaded and standard enrichment was performed using Gene Ontology and Kyoto Encyclopedia of Genes and Genomes (KEGG) Pathways. False discovery rate threshold was 0.05.
